# NS3 Resistance-Associated Variants (RAVs) in Patients Infected with HCV Genotype 1a in Spain

**DOI:** 10.1371/journal.pone.0163197

**Published:** 2016-09-29

**Authors:** María Ángeles Jimenez-Sousa, Mónica Gutiérrez-Rivas, Alejandro Álvaro-Meca, Mónica García-Álvarez, P. Richard Harrigan, Cesare Giovanni Fedele, Verónica Briz, Sonia Vázquez-Morón, Salvador Resino

**Affiliations:** 1 Viral Infection and Immunity Unit, National Centre for Microbiology. Instituto de Salud Carlos III, Majadahonda, Madrid, Spain; 2 Preventive Medicine and Public Health Unit, Faculty of Health Sciences, Rey Juan Carlos College, Alcorcón, Madrid, Spain; 3 British Columbia Centre for Excellence in HIV/AIDS, Vancouver, Canada; 4 Diagnostic Approach Area, National Centre for Microbiology, Instituto de Salud Carlos III, Majadahonda, Madrid, Spain; Saint Louis University, UNITED STATES

## Abstract

**Background:**

Resistance-associated variants have been related to treatment failure of hepatitis C virus (HCV) therapy with direct-acting antiviral drugs. The aim of our study was to analyze the prevalence of clinically relevant resistance-associated variants within NS3 in patients infected with HCV genotype 1a (GT1a) in Spain.

**Methods:**

We performed a cross-sectional study on 2568 patients from 115 hospitals throughout Spain (2014–2015). The viral NS3 protease gene was amplified by nested polymerase chain reaction and sequenced by Sanger sequencing using an ABI PRISM 377 DNA sequencer. Additionally, clade information for genotype 1a was obtained by using the software geno2pheno (http://hcv.geno2pheno.org/).

**Results:**

In total, 875 out of 2568 samples were from human immunodeficiency virus (HIV)/HCV-coinfected patients. Q80K was the main RAV found in our patients (11.1%) and the rest of the resistance-associated variants had a lower frequency, including S122G (6.23%), T54S (3.47%), V55A (2.61%), and V55I (2.15%), which were among the most frequent after Q80K. Overall, 286 samples had the Q80K polymorphism (11.1%) and 614 (23.9%) were GT1a clade I. HIV/HCV-coinfected patients had a higher frequency of Q80K and GT1a clade I than HCV-monoinfected patients (12.9% vs. 9.6% [p = 0.012] and 28.5% vs. 21.4% [p<0.001], respectively). Both the prevalence of Q80K and GT1a clade I were not uniform throughout the country (p<0.001), which ranged from 7.3%-22.2% and 15.7%-42.5%, respectively. The frequency of the Q80K polymorphism was far higher in patients infected with GT1a clade I than in patients infected with GT1a clade II (41.5% vs. 1.6%; p<0.001).

**Conclusions:**

The prevalence of most resistance-associated variants in NS3 was low in patients infected with HCV GT1a in Spain, except for Q80K (11.1%), which was also notably higher in HIV/HCV-coinfected patients. The vast majority of Q80K polymorphisms were detected in GT1a clade I.

## Introduction

Hepatitis C virus (HCV) therapy has changed rapidly with new direct-acting antiviral drugs (DAAs), particularly for HCV genotype 1, achieving high rates of sustained virologic response [1]. However, one of the main problems with new DAAs is the presence of resistance-associated variants (RAVs), which are naturally existing polymorphisms in the HCV genome that result in less susceptibility to DAAs and can lead to virological failure to HCV treatment [2]. Thus, prior knowledge of the prevalence of RAVs could be useful to determine pre-treatment management with DAAs.

HCV NS3 protease is a very attractive target for therapeutic intervention but shows a high degree of genetic variability and is able to influence HCV susceptibility to NS3 protease inhibitors (PIs) [1]. Several RAVs within NS3 protease have been described with generally a low frequency in HCV genotype 1-infected patients [3], except for the Q80K variant, which causes no loss of replicative fitness in many patients resulting in a relatively high probability of pre-existence [2]. The Q80K variant has been associated with resistance to some approved PIs (simeprevir, asunaprevir, paritaprevir) in *in-vitro* phenotypic assays [1]. In clinical trials, presence of the Q80K variant at baseline has only a significant effect on HCV treatment with simeprevir in combination with pegylated interferon alpha and ribavirin in patients infected with HCV genotype 1a (GT1a), but may also facilitate the emergence of additional HCV mutations and subsequent failure to therapy [4]. Thus, screening for Q80K is recommended before treatment with simeprevir is initiated [5].

HCV GT1a strains have been described as belonging to two distinct clades, clade I and II, which are both related to the development of antiviral resistance [6]. Interestingly, the Q80K variant is detected almost exclusively in viral isolates from patients infected with HCV GT1a, clade I [7,8]. The highest Q80K prevalence has been reported in North America where 47% of patients present this polymorphism [9]. In contrast, a lower Q80K prevalence in HCV-infected patients with GT1a has been found in European studies, varying from 5%-40% according to geographic location [10–16].

The aim of this study was to analyze the prevalence of clinically relevant RAVs within NS3 in patients infected with HCV GT1a in Spain.

## Materials and Methods

### Patients and samples

We performed a cross-sectional study in chronically infected individuals with HCV GT1a from 115 hospitals distributed geographically throughout 18 out of the 19 autonomous communities of Spain between October 2014 and October 2015. The samples were sent to the National Center of Microbiology (Instituto de Salud Carlos III [ISCIII]) for the Q80K determination, together with a minimum data set (patient code, age, gender, HIV infection, hospital, and region). These data and samples were anonymized and transferred to the ISCIII National Biobank (Ref.: B.0000984). The study was conducted in accordance with the Declaration of Helsinki. The Institutional Review Board and the Research Ethic Committee of ISCIII approved the study.

Initially, 2971 samples were used. Of those, there were 207 samples did not work or we were not able to achieve a HCV sequence consensus by Sanger sequencing. An additional 196 samples had a consensus sequence of other HCV GT1 genotypes (193 GT1b, 1 GT1i, 1 GT1e, and 1 GT1h). In total, 2568 patients with GT1a were available for statistical analysis.

### Amplification and sequencing of HCV NS3A

Viral RNA was extracted from 200μl plasma with the QIAsymphony DSP Virus/Pathogen Kit (Qiagen, Hilden, Germany).

The amplification was performed using the RT-PCR One Step kit (Qiagen, Hilden, Germany). Eight microliters of viral RNA was added to an RT-PCR mixture containing 8μl of 5X reaction buffer, 1.6μl of 10mM dNTPs, 1.2μl of 25μM HCV1NS3SF1 5’TGGAGACYAAGMTCATYACSTGGG3’ and HCV1NS3SR1 5’ACYTTRGTGCTYTTRCCGCTGCC3’ primers, 0.25μl of protector RNase inhibitor 10,000U (Roche Diagnostics GmbH), 1.6μl of Enzyme mix, and DEPC-treated water up to 40μl. Amplification was programmed as follows: 30 min at 54°C; 15 min at 95°C; 10 repetitive cycles of 30 sec at 94°C, 30 sec at 62°C (-0.5°C per cycle) and 45 sec at 72°C; 30 repetitive cycles of 30 sec at 94°C, 30 sec at 58°C, 50 sec at 72°C.

For nested PCR, 2μl of the primary amplification product was added to a mix containing 2μl of 60% sucrose-0.08% cresol red; 2μl of 10X PCR buffer 2w/15mM MgCl2; 0.8μl of 25mM MgCl2; 0.16μl of dNTPs, each at 25mM (Deoxy-NTP Set, Roche); 0.12μl of each primer at 25μM (HCV1NS3SF2 5’GAYACCGCSGCGTGYGGDGACATCA3’ and HCV1NS3SR2 5’GGGAGCRTGYAGRTGGGCCACYTGG3’); 0.29μl of expand HiFi enzyme; and DEPC-treated water up to 20μl. All reagents except primers, 60% sucrose-0.08% cresol red and dNTPs were supplied with the Roche Expand High Fidelity System kit (Roche). The thermal conditions were 3 min at 95°C; 10 repetitive cycles of 15 sec at 94°C, 30 sec at 64°C (-0.5°C per cycle), 45 sec at 72°C; followed by 30 cycles of 15 sec at 94°C, 30 sec at 58°C, 50 sec (+3sec per cycle) at 72°C. Negative and positive controls were included in all amplification procedures. PCR products were visualized on a 1% agarose gel containing 0.1μl/ml of 10,000X SYBR^®^ safe (Invitrogen). Positive samples showed a HCV specific band size of ~700 bp.

Nested amplification products were diluted 1:50 using nuclease free water (Roche). Then, the sequencing reaction was performed with 2μl of nested primers (HCV1NS3SF2 or HCV1NS3SR2 8.7μM) and 8μl of amplicon dilution previously prepared and were run on an ABI PRISM 377 DNA sequencer (Applied Biosystems, Foster City, CA).

### Bioinformatic analysis

The consensus sequences were obtained using SeqMan program (Lasergene DNASTAR Inc, Madison, WI, USA) and aligned with MEGA6 (MEGA6: Molecular Evolutionary Genetics Analysis Version 6.0; http://www.megasoftware.net/), together with representative HCV1 sequences. The NS3 gene was reviewed to determine the presence of RAVs for HCV GT1a according to the recent review of Lontok et al. [3]. Finally, the two recognized HCV-1a lineages (clade I and clade II) were identified by using the software geno2pheno (Bonn, Germany; http://hcv.geno2pheno.org/).

### Statistical analysis

The statistical analysis was performed with the Statistical Package for the IBM SPSS Statistics for Windows, Version 21.0 (IBM Corp, Chicago, Armonk, NY, USA). All p-values were two-tailed. Statistical significance was defined as p<0.05. Categorical data and proportions were analyzed using the chi-squared test or Fisher’s exact test. Multivariate logistic regression analysis was used to investigate the relationship of several factors with Q80K and clade I. The covariates included were age, gender, HIV coinfection, and original nationality.

## Results

### Characteristics of study population

Table 1 and S1 Table shows the characteristics of the 2568 HCV patients included in this study. A total of 875 of them were HIV/HCV-coinfected patients and 204 patients did not have any data on HIV status. The mean age was 49.4 years and 79.4% were male. The origin of the patients was 84.9% Spanish and 2.05% foreign, while 13.05% were of unknown origin.

**Table 1:** Epidemiological characteristics of patients included in the study.

		HIV status		
Characteristics	All patients	HCV-monoinfected	HIV/HCV-coinfected	N/A
**No.**	2568	1489 (58.0%)	875 (34.1%)	204 (7.9%)
**Age (years)**	49.4±8.3	49.5±0.2	48.9±0.2	49.9±0.5
**Gender (male)**	2041 (79.4%)	1189 (79.9%)	689 (78.7%)	163 (79.9%)
**Original nationality**				
**Spanish**	2179 (84.9%)	1321 (88.7%)	677 (77.4%)	181 (88.7%)
**Foreign**	53 (2.05%)	34 (2.3%)	13 (1.5%)	6 (2.9%)
**Unknown**	336 (13.05%)	134 (9.0%)	185 (21.1%)	17 (8.3%)

Values were expressed as absolute count (percentage) and mean ± mean standard error. Abbreviations: HCV, hepatitis C virus; HIV, human immunodeficiency virus; N/A, HIV status not available.

### Resistance-associated variants within NS3

**Fig 1:**
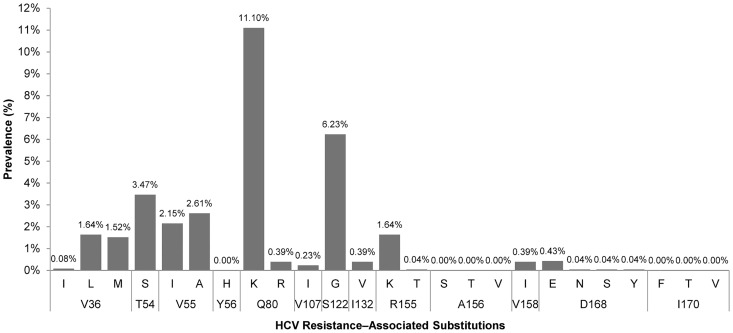
Prevalences of HCV Resistance–Associated Variants within NS3 for HCV GT1a in Spain.

The prevalences of RAVs with demonstrated clinical relevance within the NS3 gene for HCV GT1a, according to the recent review by Lontok et al. [3], are shown in (Fig 1**)**.

Q80K was the main RAV found in patients infected with HCV GT1a in Spain (11,1%) and the next most prominent RAVs were S122G (6.23%), T54S (3.47%), V55A (2.61%), and V55I (2.15%). The other variations that are described as major RAVs for currently approved PIs appeared with much lower frequencies: V36L (1.64%)/M (1.52%), R155K (1.64%), and D168E (0.43%). Because of the low frequencies found for most RAVs, Q80K was the only RAV that allowed us to perform a stratified statistical analysis with guarantee.

### Q80K polymorphism, HCV clades, and coinfection HIV/HCV

**Fig 2:**
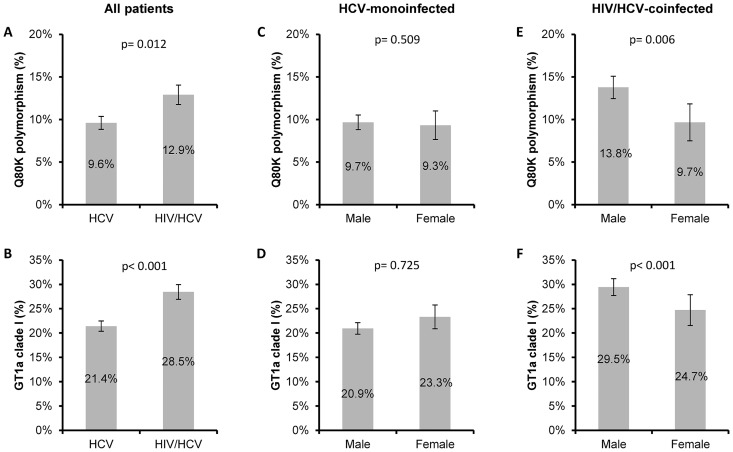
Prevalence of the Q80K polymorphism and GT1a clade I in Spain stratified by HIV coinfection and gender. (A), Prevalence of Q80K in all patients infected with HCV stratified by HIV status. (B), Prevalence of GT1a clade I in all patients infected with HCV stratified by HIV status. (C), Prevalence of Q80K in HCV-monoinfected patients stratified by gender. (D), Prevalence of GT1a clade I in HCV-monoinfected patients stratified by gender. (E), Prevalence of Q80K in HIV/HCV coinfected patients stratified by gender. (F), Prevalence of GT1a clade I in HIV/HCV coinfected patients stratified by gender. Error bars represent mean standard error. P-values were calculated by Chi-square test. Abbreviations: HCV, hepatitis C virus; HIV, human immunodeficiency virus; GT1a, HCV genotype 1a; Q80K, Q80K polymorphism (glutamine to lysine mutation) in the NS3 protein.

We found 286 (11.1%; 95% of confidence interval [95%CI] = 9.9%-12.4%) patients with the Q80K polymorphism and 614 (23.9%; 95%CI = 22.3%-25.6%) patients with GT1a clade I. HIV/HCV-coinfected patients had a higher frequency than HCV-monoinfected patients for the Q80K variant (12.9% vs. 9.6%; p = 0.012; Fig 2A) and GT1a clade I (28.5% vs. 21.4%; p<0.001; Fig 2B). We also performed a multivariate logistic regression analysis, which showed that the odds of finding Q80K or GT1a clade I were higher in HIV/HCV-coinfected patients than in HCV-monoinfected patients (adjusted odds ratio [a OR] = 1.31; [95%CI = 1.06; 1.75], p = 0.032 and a OR = 1.48 [95%CI = 1.17; 1.74], p<0.001; respectively]. On the other hand, no differences were found for the Q80K variant or clade I between males and females in HCV-monoinfected patients (Fig 2C and 2D). However, men coinfected with HIV/HCV had the greatest frequency of the Q80K polymorphism (13.8% vs. 9.7%; p = 0.006; Fig 2E) and GT1a clade I (29.5% vs. 24.7%; p<0.001; Fig 2F), but these differences were lost when the multivariate analysis was performed (*data not shown*).

**Fig 3:**
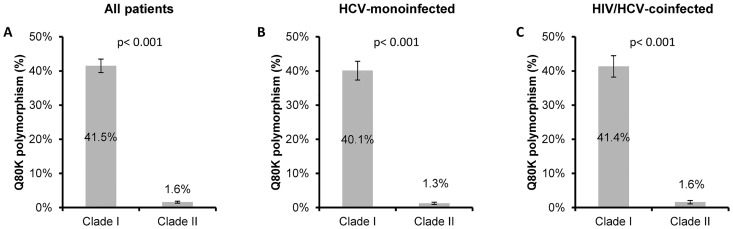
Prevalence of the Q80K polymorphism in Spain stratified by GT1a clades. (A), Prevalence of Q80K in all patients infected with HCV stratified by GT1a clade I. (B), Prevalence of Q80K in HCV-monoinfected patients stratified by GT1a clade I. (C), Prevalence of Q80K in HIV/HCV coinfected patients stratified by GT1a clade I. Error bars represent mean standard error. P-values were calculated by Chi-square test. Abbreviations: HCV, hepatitis C virus; HIV, human immunodeficiency virus; GT1a, HCV genotype 1a; Q80K, Q80K polymorphism (glutamine to lysine mutation) in the NS3 protein.

Moreover, the frequency of the Q80K polymorphism was higher in patients infected with GT1a clade I than in patients infected with GT1a clade II (41.5% vs. 1.6%; p<0.001; Fig 3A). When we analyzed the distribution of the Q80K polymorphism regarding GT1a clades, HCV-monoinfected patients and HIV/HCV-coinfected patients also had higher frequency of the Q80K polymorphism in GT1a clade I than GT1a clade II (Fig 3B and 3C). However, note that statistically significant differences were not found in Q80K prevalence between HIV/HCV-coinfected and HCV-monoinfected patients within clade I (41.4% vs. 40.1%; p = 0.765) or clade II (1.6% vs. 1.3%; p = 0.587) (Fig 3B and 3C). In multivariate logistic regression analysis, the odds of finding Q80K were higher in patients infected with GT1a clade I than in patients infected with HCV GT1a clade II (a OR = 46.43 [95%CI = 31.26; 68.93], p<0.001).

### Distribution of the Q80K polymorphism and GT1a clade I in Spain

The prevalences of the Q80K polymorphism and GT1a clade I distributed throughout the different regions of Spain were not uniform throughout the country (p<0.001), and it ranged from 7.3%- to 22.2% and 15.7% to 42.5%, respectively Table 2.

**Table 2:** Distribution of the Q80K polymorphism and GT1a clade I in HCV-infected patients throughout regions (autonomous communities) of Spain.

		Q80K polymorphism		GT1a clade I	
Regions	All	No.	Crude prevalence	No.	Crude prevalence
**Andalusia**	174	16	9.2%	39	22.4%
**Aragon**	39	6	15.4%	11	28.2%
**Asturias**	142	14	9.9%	32	22.5%
**Balearic Islands**	40	5	12.5%	7	17.5%
**Basque country**	424	38	9.0%	73	17.2%
**Canary Islands**	106	21	19.8%	45	42.5%
**Cantabria**	117	12	10.3%	33	28.2%
**Castile-La Mancha**	19	2	10.5%	5	26.3%
**Castille and Leon**	240	21	8.8%	58	24.2%
**Catalonia**	94	10	10.6%	16	17.0%
**Ceuta**	9	2	22.2%	3	33.3%
**Extremadura**	53	9	17.0%	13	24.5%
**Galicia**	540	52	9.6%	139	25.7%
**La Rioja**	37	3	8.1%	8	21.6%
**Madrid**	229	48	21.0%	77	33.6%
**Murcia**	30	5	16.7%	10	33.3%
**Navarre**	97	9	9.3%	17	17.5%
**Valencian community**	178	13	7.3%	28	15.7%

Values were expressed as absolute count and percentage. Abbreviations: GT1a, HCV genotype 1a; Q80K, Q80K polymorphism (glutamine to lysine mutation) in the NS3 protein.

## Discussion

There exists little data on the prevalence of NS3 RAVs in large cohorts of HCV patients from particular geographical areas in Europe. In our study, the prevalence of NS3 RAVs was low except for Q80K. Our results are in the same range as those reported in a treatment-naive population in Europe [4].

Overall, the impact of most NS3 RAVs on HCV DAA treatment in patients infected with HCV GT1a should be low because of its low prevalence. The exception to this is Q80K, which occurred in 11.1% of the sample population examined here. The other most frequent RAVs were S122G (6.23%), T54S (3.47%), V55A (2.61%), and V55I (2.15%), which have a low clinical impact in HCV GT1a–infected patients who are treated with NS3 inhibitors [3]. The concurrence of Q80K and V36L/M, R155K, D168E when starting HCV therapy may confer significant resistance to treatment regimens containing simeprevir even when being treated with other potent DAAs [17]. Therefore, we also searched for these RAVs and found a very lower frequency for V36L (1.64%)/M(1.52%), R155K (1.64%), D168E (0.43%) and several other associations such as Q80K/R155K (0.12%) and V36M/R155K (0.45%), which have been described to confer high viral fitness [2]. To date, only a few articles describing the prevalences of Q80K and GT1a clade I have been published since the introduction of DAAs [8,11–16]. In our study, the reported prevalences comprised a large dataset from 2568 patients infected with GT1a from 18 regions in Spain, which contributes to the robustness to our data. The observed Q80K prevalence was 11.1%, which was higher than that initially described by Sarrazin et al. [16], but similar to what has been found in Spain’s neighboring European countries (Portugal and France) [16]. However, the observed prevalence for GT1a clade I was 24.1% (614/2568), which was substantially lower than a previously described European cohort (49.3%) [8] and a French cohort (43.2%) [13].

Our data show that the prevalence of Q80K and GT1a clade I in our patients varied regarding to HIV status. We found that the likelihood of finding Q80K and GT1a clade I were significantly higher in HIV/HCV-coinfected patients in multivariate regression. Some previously reported data show a discrepancy in Q80K prevalence in coinfected versus monoinfected patients [18], but other reports did not find any differences in prevalence of Q80K and/or GT1a clade I between these two patient subsets [11,19–22]. These discrepancies could be explained by the limited sample sizes used in previous studies, for example when the prevalence data of Q80K in HIV/HCV-coinfected patients have been generated from small sample sizes in large territories. However, in our study we included a large number of patients (n = 2568), of which 34% (n = 875) were HIV/HCV-coinfected. Although the number of patients with Q80K is low (n = 235), these are the data that we have found in a large sample size. To our knowledge, our study used the largest published sample size from one country, which is a major advantage for drawing a valid conclusion. The differences found between HIV/HCV-coinfected and HCV-monoinfected subjects might be related to differences in transmission of HIV and HCV, such as sexual risk behaviors or drug use behavior [23,24]. Further research is needed to confirm this pattern of Q80K distribution in patients coinfected with HIV/HCV.

The prevalence of Q80K and GT1a clade I show a marked geographical variability in Europe [10,11,13,14,16]. Regarding Spain, our study shows that the geographical distribution of Q80K and GT1a clade I was also not uniform, with differences up to 15% and 18% for the prevalence of Q80K and GT1a clade I, respectively, among regions. Therefore, although the prevalence ranges could be considered normal for European countries [8,10,11,13,14,16], it is not advisable to consider the prevalence of Q80K to be valid when small sample sizes have been used and it has not been compared across regions.

Interestingly, the Q80K prevalence has been linked to the circulation of two distinct GT1a lineages (North America [clade I] and Europe [clade II]) with and without Q80K, respectively [6,25]. Clade I is more frequent in GT1a patients from the United States compared to those from Europe, which is consistent with the higher prevalence of Q80K in GT1a in the United States [8]. In our study, the vast majority of occurrences of the Q80K polymorphism was detected in patients with GT1a clade I. However, the presence of the Q80K polymorphism was not exclusive to GT1a clade I, as it was present in a few patients with HCV belonging to GT1a clade II (1.6% (31/1954). This varies from previous reports, which have mostly described the absence of Q80K detection in GT1a clade II [8,10,11]. To our knowledge, there is only one recent article describing the presence of Q80K among GT1a clade II, showing a prevalence of around 13% (9/69) [18], which is much higher than that obtained in our study. These conflicting results could be due to a limited sample size, indicating that this seems to be the major limitation to reaching any robust significant conclusions. It is possible that the presence of Q80K in GT1a clade II could represent a small percentage of recombinant viruses from both clades I and II, which would break the premise that Q80K is uniquely associated with clade I [8]. Further studies are needed to confirm the possibility of recombination among viruses from clades I and II.

This study has relevant limitations, some of which have been discussed above: (i) the number of patients found exhibiting baseline RAVs (not including Q80K) was quite low and does not allow us to draw strong conclusions for some RAVs; (ii) the association of RAVs with SVR data were not assessed in this study, but the clinically relevant RAVs within NS3 were extrapolated from a recent review by Lontok et al. [3]; (iii) we used Sanger sequencing as a detection method (population sequencing), which is less sensitive for RAVs detection than deep sequencing; however, the need for a rapid Q80K diagnosis may dispute deep sequencing as an option due to higher turnaround time and cost. Moreover, no recommendations were available for this purpose; (iv) access to epidemiological and clinical data, such HCV transmission mode or HCV viral load, was limited, thus we were unable to perform multivariate analysis including these variables.

## Conclusions

In conclusion, the prevalence of NS3 RAVs was low in patients infected with HCV GT1a in Spain, except for Q80K (11%), which was higher in HIV/HCV-coinfected patients. The vast majority of the instances of the Q80K polymorphism was detected in GT1a clade I, but it was also detected in GT1a clade II.

## Supporting Information

S1 Table Minimum data set underlying the study findings contained in the manuscript. (DOCX)

## References

[1] Gutierrez JA, Lawitz EJ, Poordad F (2015) Interferon-free, direct-acting antiviral therapy for chronic hepatitis C. J Viral Hepat 22: 861–870. 10.1111/jvh.12422 26083155

[2] Sarrazin C (2016) The importance of resistance to direct antiviral drugs in HCV infection in clinical practice. J Hepatol 64: 486–504. 10.1016/j.jhep.2015.09.011 26409317

[3] Lontok E, Harrington P, Howe A, Kieffer T, Lennerstrand J, Lenz O, et al (2015) Hepatitis C virus drug resistance-associated substitutions: State of the art summary. Hepatology 62: 1623–1632. 10.1002/hep.27934 26095927

[4] Lenz O, Verbinnen T, Fevery B, Tambuyzer L, Vijgen L, Peeters M, et al (2015) Virology analyses of HCV isolates from genotype 1-infected patients treated with simeprevir plus peginterferon/ribavirin in Phase IIb/III studies. J Hepatol 62: 1008–1014. 10.1016/j.jhep.2014.11.032 25445400

[5] (2014) EASL Clinical Practice Guidelines: management of hepatitis C virus infection. J Hepatol 60: 392–420. 10.1016/j.jhep.2013.11.003 24331294

[6] Pickett BE, Striker R, Lefkowitz EJ (2011) Evidence for separation of HCV subtype 1a into two distinct clades. J Viral Hepat 18: 608–618. 10.1111/j.1365-2893.2010.01342.x 20565573 PMC2964416

[7] Bagaglio S, Uberti-Foppa C, Messina E, Merli M, Hasson H, Andolina A, et al (2015) Distribution of natural resistance to NS3 protease inhibitors in hepatitis C genotype 1a separated into clades 1 and 2 and in genotype 1b of HIV-infected patients. Clin Microbiol Infect 10.1016/j.cmi.2015.12.007 . 26706617

[8] De Luca A, Di Giambenedetto S, Lo Presti A, Sierra S, Prosperi M, Cella E, et al (2015) Two Distinct Hepatitis C Virus Genotype 1a Clades Have Different Geographical Distribution and Association With Natural Resistance to NS3 Protease Inhibitors. Open Forum Infect Dis 2: ofv043 10.1093/ofid/ofv043 26213689 PMC4511743

[9] Bae A, Sun SC, Qi X, Chen X, Ku K, Worth A, et al (2010) Susceptibility of treatment-naive hepatitis C virus (HCV) clinical isolates to HCV protease inhibitors. Antimicrob Agents Chemother 54: 5288–5297. 10.1128/AAC.00777-10 20855726 PMC2981235

[10] Nguyen LT, Gray E, Dean J, Carr M, Connell J, De Gascun C, et al (2015) Baseline prevalence and emergence of protease inhibitor resistance mutations following treatment in chronic HCV genotype 1-infected individuals. Antivir Ther 10.3851/IMP2964 . 25920764

[11] Beloukas A, King S, Childs K, Papadimitropoulos A, Hopkins M, Atkins M, et al (2015) Detection of the NS3 Q80K polymorphism by Sanger and deep sequencing in hepatitis C virus genotype 1a strains in the UK. Clin Microbiol Infect 21: 1033–1039. 10.1016/j.cmi.2015.07.017 26232533

[12] Shepherd SJ, Abdelrahman T, MacLean AR, Thomson EC, Aitken C, Gunson RN (2015) Prevalence of HCV NS3 pre-treatment resistance associated amino acid variants within a Scottish cohort. J Clin Virol 65: 50–53. 10.1016/j.jcv.2015.02.005 25766988 PMC4728298

[13] Morel V, Duverlie G, Brochot E (2014) Patients eligible for treatment with simeprevir in a French center. J Clin Virol 61: 149–151. 10.1016/j.jcv.2014.06.023 25027573

[14] Vicenti I, Rosi A, Saladini F, Meini G, Pippi F, Rossetti B, et al (2012) Naturally occurring hepatitis C virus (HCV) NS3/4A protease inhibitor resistance-related mutations in HCV genotype 1-infected subjects in Italy. J Antimicrob Chemother 67: 984–987. 10.1093/jac/dkr581 22258932

[15] Leggewie M, Sreenu VB, Abdelrahman T, Leitch EC, Wilkie GS, Klymenko T, et al (2013) Natural NS3 resistance polymorphisms occur frequently prior to treatment in HIV-positive patients with acute hepatitis C. AIDS 27: 2485–2488. 10.1097/QAD.0b013e328363b1f9 23770494

[16] Sarrazin C, Lathouwers E, Peeters M, Daems B, Buelens A, Witek J, et al (2015) Prevalence of the hepatitis C virus NS3 polymorphism Q80K in genotype 1 patients in the European region. Antiviral Res 116: 10–16. 10.1016/j.antiviral.2015.01.003 25614456

[17] Vidal LL, Soares MA, Santos AF (2016) NS3 protease polymorphisms and genetic barrier to drug resistance of distinct hepatitis C virus genotypes from worldwide treatment-naive subjects. J Viral Hepat 10.1111/jvh.12503 . 26775769

[18] Ruggiero T, Proietti A, Boglione L, Milia MG, Allice T, Burdino E, et al (2015) Predominance of hepatitis C virus Q80K among NS3 baseline-resistance-associated amino acid variants in direct-antiviral-agent-naive patients with chronic hepatitis: single-centre experience. Arch Virol 10.1007/s00705-015-2563-3 . 26249823

[19] Jabara CB, Hu F, Mollan KR, Williford SE, Menezes P, Yang Y, et al (2014) Hepatitis C Virus (HCV) NS3 sequence diversity and antiviral resistance-associated variant frequency in HCV/HIV coinfection. Antimicrob Agents Chemother 58: 6079–6092. 10.1128/AAC.03466-14 25092699 PMC4187920

[20] Ehret R, Neifer S, Walter H, Baumgarten A, Obermeier M (2014) Appearance of NS3 Q80K mutation in HCV genotype 1a mono- or HIV/HCV co-infected patients in a Berlin laboratory. J Int AIDS Soc 17: 19741 10.7448/IAS.17.4.19741 25397486 PMC4225327

[21] Cao Y, Zhang Y, Bao Y, Zhang R, Zhang X, Xia W, et al (2015) Naturally occurring HCV protease inhibitors resistance-associated mutations among chronic hepatitis C genotype 1b patients with or without HIV coinfection. Hepatol Res. 10.1111/hepr.12590 26355704

[22] Lisboa-Neto G, Noble CF, Pinho JR, Malta FM, Gomes-Gouvea MS, Alvarado-Mora MV, et al (2015) Resistance mutations are rare among protease inhibitor treatment-naive hepatitis C genotype-1 patients with or without HIV coinfection. Antivir Ther 20: 281–287. 10.3851/IMP2873 25279715

[23] Bradshaw D, Matthews G, Danta M (2013) Sexually transmitted hepatitis C infection: the new epidemic in MSM? Curr Opin Infect Dis 26: 66–72. 10.1097/QCO.0b013e32835c2120 23242342

[24] Parikh N, Nonnemacher MR, Pirrone V, Block T, Mehta A, Wigdahl B (2012) Substance abuse, HIV-1 and hepatitis. Curr HIV Res 10: 557–571. 22973853 10.2174/157016212803306023 PMC3708479

[25] McCloskey RM, Liang RH, Joy JB, Krajden M, Montaner JS, Harrigan PR, et al (2015) Global origin and transmission of hepatitis C virus nonstructural protein 3 Q80K polymorphism. J Infect Dis 211: 1288–1295. 10.1093/infdis/jiu613 25389307

